# Supplementary material for: Polyvinyl Alcohol-Few Layer Graphene Composite Films Prepared from Aqueous Colloids. Investigations of Mechanical, Conductive and Gas Barrier Properties
Source: Nanomaterials (Basel). 2020 Apr 29;10(5):858. doi: 10.3390/nano10050858 (PMC7711457; doi:10.3390/nano10050858)
Supplement: Supplementary file 1 [file nanomaterials-10-00858-s001.pdf]

Supplementary Materials:
Polyvinyl Alcohol-Few Layer Graphene Composite Films Prepared from Aqueous Colloids. Investigations of Mechanical, Conductive and Gas Barrier Properties

Benoit Van der Schueren [1], Hamza El Marouazi [1], Anurag Mohanty [1], Patrick Lévêque [2], Christophe Sutter [1], Thierry Romero [1] and Izabela Janowska [1,*]

[1] Institut de Chimie et Procédés pour l'Énergie, l'Environnement et la Santé (ICPEES), CNRS UMR 7515-University of Strasbourg, 25 rue Becquerel, 67087 Strasbourg, France; benelux88@gmail.com (B.V.S.); hamza.el-marouazi@etu.unistra.fr (H.E.M.); anurag.mohanty@etu.unistra.fr (A.M.); christophe.sutter@unistra.fr (C.S.); thierry.romero@unistra.fr (T.R.)

[2] Laboratoire des sciences de l'Ingénieur, de l'Informatique et de l'Imagerie (ICube), UMR 7357, CNRS, Université de Strasbourg, 67400 Strasbourg, France; patrick.leveque@unistra.fr

* Correspondence: janowskai@unistra.fr

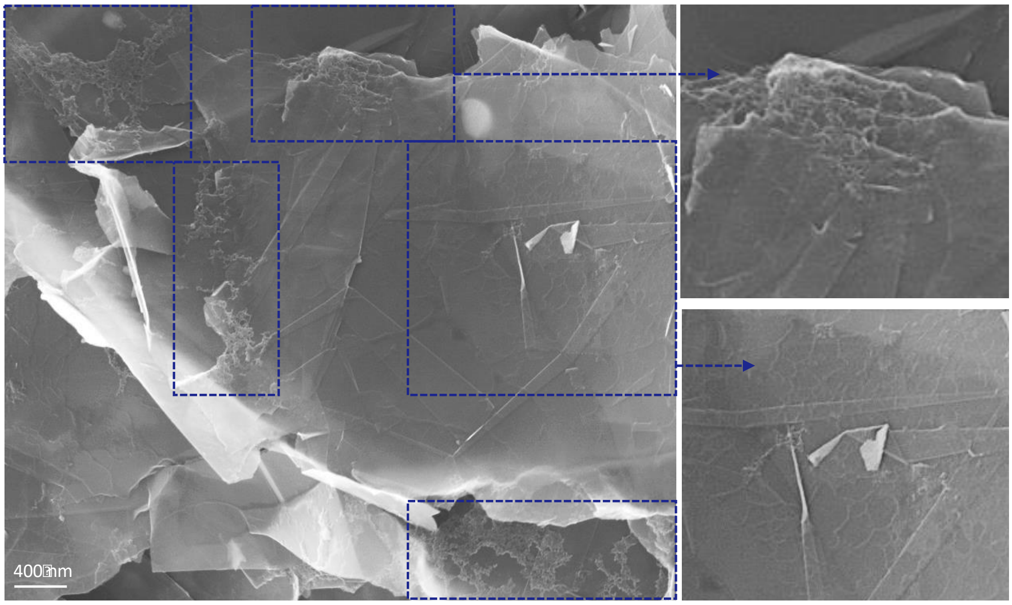

Figure S1. SEM micrograph of FLG flakes' aggregate with visible BSA chains adsorbed over the surface (right down) and the edges (right top) of the flakes.

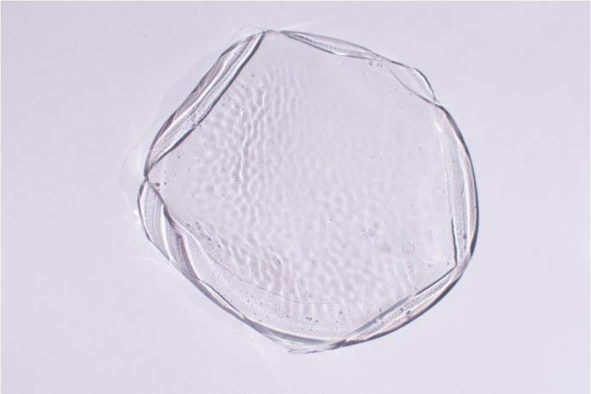

Figure S2. Optical image of PVA film with peculiar wavy network aspect.

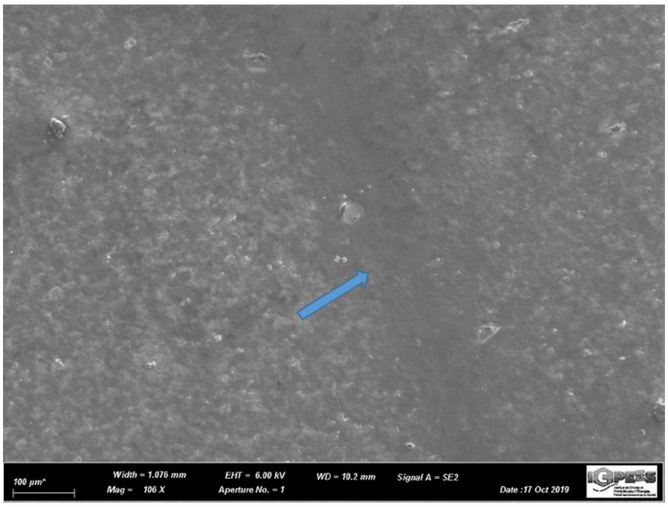

Figure S3. SEM micrograph of the surface of PVA-FLG film containing macroscopic network of FLG. One can see, indicated by the arrow, one of the branches from the network filled with FLG flakes.
